# Vertebrate growth plasticity in response to variation in a mutualistic interaction

**DOI:** 10.1038/s41598-022-14662-4

**Published:** 2022-07-18

**Authors:** Theresa Rueger, Anjali Kristina Bhardwaj, Emily Turner, Tina Adria Barbasch, Isabela Trumble, Brianne Dent, Peter Michael Buston

**Affiliations:** 1 Department of Biology and Marine Program, Boston University, Boston, MA USA; 2 College of Life Environmental Sciences, University of Exeter, Penryn, UK; 3 Dove Marine Laboratory, School of Natural and Environmental Sciences, Newcastle University, Newcastle Upon Tyne, UK

**Keywords:** Behavioural ecology, Social evolution

## Abstract

Vertebrate growth can be phenotypically plastic in response to predator–prey and competitive interactions. It is unknown however, if it can be plastic in response to mutualistic interactions. Here we investigate plasticity of vertebrate growth in response to variation in mutualistic interactions, using clown anemonefish and their anemone hosts. In the wild, there is a positive correlation between the size of the fish and the size of the anemone, but the cause of this correlation is unknown. Plausible hypotheses are that fish exhibit growth plasticity in response to variation in food or space provided by the host. In the lab, we pair individuals with real anemones of various sizes and show that fish on larger anemones grow faster than fish on smaller anemones. By feeding the fish a constant food ration, we exclude variation in food availability as a cause. By pairing juveniles with artificial anemones of various sizes, we exclude variation in space availability as a single cause. We argue that variation in space availability in conjunction with host cues cause the variability in fish growth. By adjusting their growth, anemonefish likely maximize their reproductive value given their anemone context. More generally, we demonstrate vertebrate growth plasticity in response to variation in mutualistic interactions.

## Introduction

A fundamental question of evolutionary ecology is, what determines body size? While the growth and size of vertebrates is thought to be relatively fixed compared to that of invertebrates, plasticity of vertebrate growth and size has been known to occur in response to a wide variety of both abiotic and biotic conditions^1^. Abiotic conditions and ecological interactions (competition and predation) have all been known to elicit plasticity of vertebrate growth^1–5^. Understanding this plasticity of growth is essential for understanding relationships between organisms and their biotic and abiotic environments^6^. Changes in growth rate are not always a simple consequence of changing energetics (e.g., temperature or resource availability), but rather growth rate can also be adjusted in anticipation of changing circumstances. For example, shrews of the genus *Sorax* change their skull sizes in anticipation of seasonal changes^7,8^, crucian carp (*Carassius carassius*) increase their body depth when in the presence of their predator, piscivorous pike (*Esox lucius*)^2^; and, Kalahari meerkats (*Suricata suricatta*) increase their growth rates when the growth rate of reproductive competitors is artificially increased^5^. To our knowledge, there are no documented examples of vertebrate growth plasticity in response to variation in mutualistic interactions.

Mutualisms are species interactions where the inclusive fitness of each party is increased through actions of its partner^9^. Mutualisms are common, crossing all domains of life and playing a central role in ecosystem evolution^10^. Many mutualistic partners are size matched in the wild and often the apparent good fit between one partner and the other may be adaptive. For example, fig pollinator wasps cannot grow too large as to not fit through the fig’s ostiole, even though larger wasps are more successful disperses^11^. Similarly, extinct large-bodied frugivores were size matched to the large seeds they dispersed over long-distances^12^. While there are examples of invertebrates, vertebrates, and plants evolving to match each other’s size^13,14^, there are no examples of plasticity in response to mutualism.

A tractable system to test whether vertebrate growth can be plastic in response to a mutualistic partner is the well-known mutualism between anemonefishes of the genus *Amphiprion* and their cnidarian host^15–17^. Anemonefishes occupy anemones in groups of 2–13 individuals, organized in size-based dominance hierarchies^18,19^. The anemone provides the fish and their eggs with protection from predators^20,21^, and the fish provide the anemone with protection from predators too^15^. Nutrients are also transferred from the host anemone and symbiotic zooxanthellae to the anemonefish^22^, and from anemonefish to the host^23^. Across the *Amphiprion* genus, there is a widespread correlation between anemone size and fish size, both the size of the dominant individual and the summed standard lengths of the whole group^15,19,24–28^. It is possible that the correlation between fish and anemone size is a simple product of variation in food or space availability or that the fish can regulate their growth in an anticipatory fashion to maximize their reproductive value on a given anemone.

Here, we investigated whether vertebrate growth can be plastic in response to variation in the size of a mutualistic partner. We used the clown anemonefish, *Amphiprion percula*, which has been shown to exhibit growth plasticity in response to competition during social group formation and maintenance^19,29^. In *A. percula*, the correlation between host size and fish size is unlikely to be a result of variation in mortality since mortality rates of *A. percula* do not vary with anemone size^30^. Also, *A. percula* do not switch anemone hosts once they are settled^31^, excluding the possibility of larger fish usurping larger hosts. In this study we first tested the hypothesis that the standard length of dominant rank one *A. percula* is correlated with the size of their anemone host, using data from a wild population in Papua New Guinea. Second, we tested the hypothesis that anemonefish growth will be positively correlated with anemone size, using real anemones in the laboratory. Third, we tested alternative hypotheses for the cue of the observed effect (food and space availability), using fake anemones in the laboratory. We found that vertebrate growth can indeed be plastic in response to variation in a mutualistic interaction and we found that space availability together with a biological cue from the host are the most likely trigger for growth plasticity in the anemonefish-anemone mutualism.

## Materials and methods

### Field measurements

To test the correlation between anemone size and the size of *A. percula* (hypothesis 1), we conducted a field study in November and December 2019 on inshore reefs near Mahonia Na Dari Research and Conservation Centre in Kimbe Bay, Papua New Guinea. We located 202 groups of *Amphiprion percula* on *Heteractis magnifica* on 15 reefs. All individual fish in each group were caught using hand nets and their standard length was measured using calipers to the nearest 0.1 mm*.* Rank was determined based on size, as this species maintains a strict size ratio between ranks^19^. The fish were returned to their host following measurement. Anemone oral disks were measured using soft tailors’ tape, measuring to the nearest cm from tentacle tip to tentacle tip on the longest diameter and again from tentacle tip to tentacle tip on the diameter perpendicular to the longest diameter. To account for expansion behavior, anemones were measured three times on three different days and the measurements were averaged. Anemone area was calculated as the area of an ellipse (A = π*a*b, with A anemone area in cm^2^, a the major axis and b the minor axis). The study is reported according to ARRIVE guidelines.

### Lab experiments

To test whether anemonefish growth is positively related to anemone size (hypothesis 2) and whether this is a response to space availability (hypothesis 3), experiments were conducted at Boston University (Boston, MA, USA) from January 2020 to June 2021 using the clown anemonefish, *Amphiprion percula*. Experimental protocols were approved by Boston University’s Institutional Animal Care and Use Committee (protocol number 17-001). All fish used in this experiment were reared from fish that were caught as non-breeders (less than 30 mm in standard length) in Papua New Guinea and supplied by Quality Marine and Sea Dwelling Creatures. All breeding pairs were paired in the lab and had been breeding for more than 5 years. A detailed description of broodstock housing conditions can be found elsewhere^32,33^. Fish used in the experiment were offspring of these breeding pairs and were reared for 28 days (±1 day), at which time they were moved into juvenile housing.

#### Juvenile housing

All experimental juvenile fish for each of the two experiments were housed in separate 113.5 L tanks with identical set up. Each tank had a 15 cm^2^ ceramic tile, with a reef rock, a few anemones (*Entacmaea quadricolor*), and a breeding pair of clownfish. At the opposite end of the tank, at a distance of approximately 20 cm, we placed a mesh cylinders (0.16 or 0.32 cm mesh), with a radius of 10.16 cm and a height of 30.5 cm, that exceeded the water level (~ 27 cm), creating a segregated space for our experimental juveniles and their anemones. Fish were introduced and covered by a 40 oz food storage container with drilled in holes covered by mesh until they settled into their anemone. Introduction chambers were removed after 24 h, leaving the fish free to move about the cylinders, though mostly they stayed nestled in their anemone tentacles.

#### Experiment 1

To test whether anemonefish growth is positively related to anemone size (hypothesis 2), we placed similar sized fish into anemones (*Entacmaea quadricolor*) of varying sizes*.* A single juvenile clownfish and one anemone were paired haphazardly, so that initial fish size was unrelated to initial anemone size. Experiment 1 was conducted over six months between January and June 2020 (n_initial_ = 44, n_end_ = 25). Experimental juvenile fish were from two sets of parents.

To rule out the possibility that variation in growth is a response to variation in food availability in anemones of different sizes, as might be the case in the wild, the amount of food that the fish received was controlled: fish were fed to satiation using a rationed diet of 0.25 ml of granulated pellets dressed with *Haematococcus*, once a day, seven days a week. Subsequently the size of anemones and growth of fish was monitored each month for six months.

#### Experiment 2

To test whether anemonefish growth is simply a response to the variation in space provided by the anemone tentacles (hypothesis 3), we placed similar sized fish into artificial anemones of varying sizes. Experiment 2 was conducted over six months between September 2020 and June 2021 (n_initial_ = 87, n_end_ = 24). Since the mortality of juvenile fish in artificial anemones was higher than in experiment 1 (see Supplemental Material and Results), juveniles from six clutches, i.e. six separate parent pairs, were used in experiment 2.

The artificial anemones were made of silicone with a weighted base (Tetra Blooming Collection Anemone Aquarium Decor, White). We manipulated the size of artificial anemones, to have 10 sizes by cutting the artificial tentacles to match the sizes of the real anemones at the start of experiment 1. The feeding regime was the same as in experiment 1 above. If juveniles died, they were removed from the tanks along with their anemones and housing units. Their data were only used until the previous month measured.

#### Anemone metrics

Anemone measurements for real anemones were made four times per month, at one-week intervals, over the course of the experiment. Artificial anemones were measured each month to account for possible wear of the silicone. Photographs were taken using an underwater camera (GoPro Hero7) from a position that maximized visible tentacle breadth including a 1 × 1 inch card for scale. ImageJ^34^ software was used to trace the perimeter of all live tissue (or equivalent in the case of artificial anemones) and estimate surface area of the anemone. Multiple measures of each anemone were taken to account for expansion behavior and growth across the month, and the average of these measures was used as an estimate of anemone size each month. Two real anemones fissioned during the study, and they were removed from the dataset for all subsequent months.

#### Fish metrics

Fish measurements were made seven times, at four-week intervals, over the course of both experiments (t_0_, t_1_, t_2_, t_3_, t_4_, t_5_, t_6_). To measure them, fish were removed from their tank and placed in a small cylinder filled with tank water for transport. They were then picked up from the cylinder using a plastic spoon and briefly placed on a microscope slide with an engraved 1 mm gridline system as a scale. The fish were photographed laterally using a stereoscopic microscope (Nikon SMZ745: 7.5 × zoom and 115 mm working distance) with a camera (Canon EOS60D). ImageJ was used to determine the standard length (SL; length measured from the distance between the tip of the snout to the last vertebrae) to 0.01 mm from each photograph three times. The average of the three measurements for SL were used as the monthly measurement for each individual. After being placed back in their cylinders, all fish swam directly to their anemones. Fish growth per month was calculated as the difference in size between two time points.

### Statistical analysis

All statistical analyses were conducted using R version 3.6.1^35^. To test the correlation between anemone size and fish size in the wild we used Pearson’s correlation coefficient. To test the hypothesis that fish growth will be a plastic response to the size of the anemone we conducted Bayesian mixed model analyses on data from experiment 1 and 2 using the *rstanarm* package weakly informative default priors^36^. We used fish growth per month (change in SL) as the dependent variables and average anemone size for each month as the independent variable of primary interest. Average anemone size each month was ln (log_e_) transformed to improve model fit. We controlled for the effect of initial fish size at the beginning of each month, because fish growth is often negatively correlated with size^37^. Fish ID was used as a random effect to account for repeated measures of the same individuals and non-independence of data points. Fit of the models was checked using the *shinystan* package^38^. Bayesian R^2^ was calculated using the *performance* package^39,40^. Model plots were produced using the *bayesplot* package^41,42^. Diagnostic plots for each model can be found in the supplementary material (suppl. Figure 2–7). We also tested the interaction between the fixed factors by conducting a log likelihood test between the model with the interaction and the model without the interaction.

## Results

### Size correlation in the wild (hypothesis 1)

**Figure 1:**
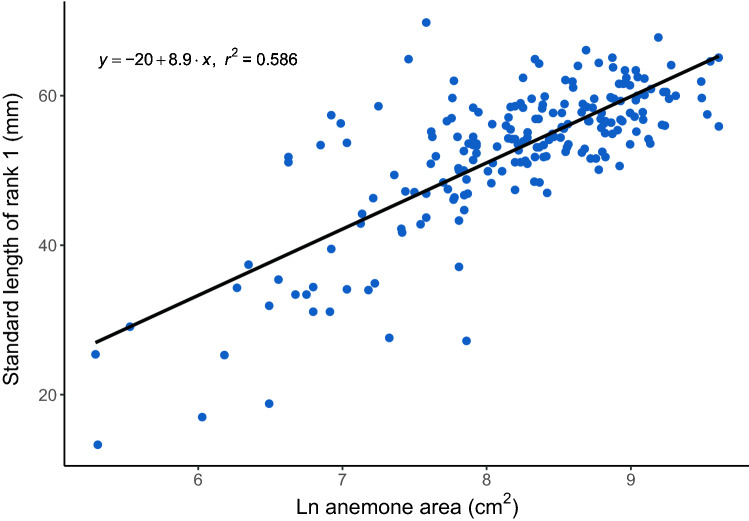
Relationship between the standard length of rank 1 *A. percula* (mm) and the size (ln cm^2^) of their *H. magnifica* host for 202 groups in Kimbe Bay, Papua New Guinea.

The standard length of rank 1 *A. percula* was positively correlated with the size of their anemone host in the wild (*H. magnifica*) (Fig. 1, Pearson’s r = 0.765, t = 16.816, *p* < 0.001).

### Growth plasticity (Hypotheses 2 and 3)

At the start of experiment 1, the mean (±SE) standard length (SL) of juvenile *A. percula* was 10.86 ± 0.19 mm, the mean (± standard error (SE)) anemone size (*E. quadricolor*) was 12.32 ± 1.44 cm^2^ (range 0.96–46.32 cm^2^), and initial fish size was unrelated to initial anemone size (Pearson Correlation: r = −0.089, *p* = 0.563).

**Figure 2:**
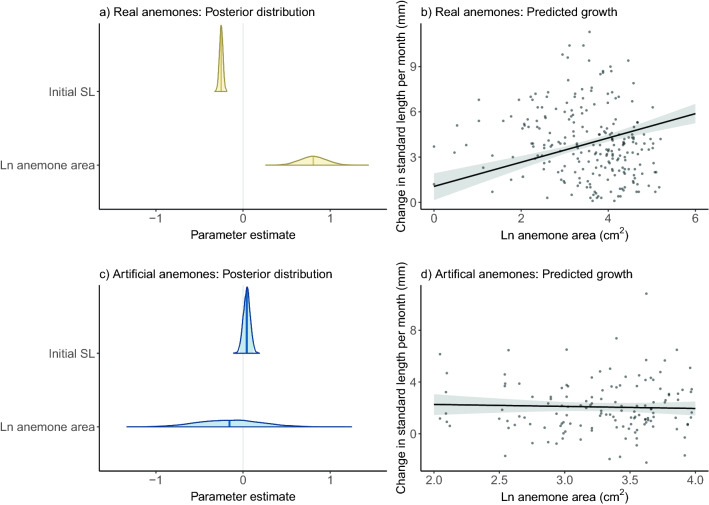
Results for two Bayesian linear mixed models testing the effect of initial standard length (SL) and Ln anemone area on growth of *Amphiprion percula*: (**a**) Posterior distributions with medians and 95% intervals for experiment 1, where fish were paired with *Entacmaea quadricolor*; (**b**) Posterior distributions with medians and 95% intervals for experiment 2, where fish were paired with artificial anemones; (**c**) Predicted change in standard length (mm) dependent on Ln anemone area (cm^2^) for *A. percula* on *E. quadricolor*; (**d**) Predicted change in standard length (mm) dependent on Ln anemone area (cm^2^) for *A. percula* on artificial anemones.

The size of real anemones in the laboratory had a significant positive effect on fish growth per month (beta = 0.81, standard deviation (SD) = 0.15, 95% credible interval (CI) [0.51, 1.11]; Fig. 2a, b). A fish on an anemone that was 50% larger grew 0.33 mm more per month on average. Initial standard length had a significant negative effect on fish growth, with larger fish growing slower (beta = −0.25, SD = 0.02, 95% CI [−0.29, −0.21]; Fig. 2a). The fixed effects explained 41% of variation in the data (Bayes R^2^ marginal: 0.41, 89% CI 0.339, 0.478).

At the start of the experiment 2 the mean (±SE) SL of the juveniles was 9.63 ± 0.21 mm, the mean (±SE) of artificial anemone size was 28.26 ± 2.02 cm^2^ (range 6.89–53.09) and initial fish size was unrelated to initial anemone size (Pearson Correlation: r = −0.022, *p* = 0.811).

The size of artificial anemones had no effect on fish growth (beta =  −0.16, SD = 0.37, 95% CI [−0.87, −0.58]; Fig. 2b, d). Initial standard length had a slight positive effect on fish growth (beta = 0.04, SD = 0.04, 95% CI [−0.04, −0.12]; Fig. 2b). The fixed effects explained 4% of variation in the data (Bayes R^2^ marginal: 0.038, 89% CI 0.000016, 0.086). Not only did fish in artificial anemones not exhibit a plastic response to anemone size, but their demographic rates were different in other ways too. Survival was much lower for fish in artificial anemones compared to those paired with real anemones (suppl. Figure 1a). Growth was much lower and more variable for fish in artificial anemones compared to those paired with real anemones (suppl. Figure 1b).

## Discussion

Our experiments showed that vertebrate growth can be plastic in response to variation in a mutualistic interaction. In one of the most well-studied marine mutualisms between fish of the genus *Amphiprion* and tropical sea anemones, there is a well-documented positive correlation between host anemone size and the size of the largest fish^16,19,28,43^. We confirmed this relationship for a wild population of *Amphiprion percula* in Kimbe Bay, Papua New Guinea. *Amphiprion percula* is the most site restricted member of the genus and rarely strays beyond the periphery of their anemone’s tentacles^44,45^, so the correlation could be a result of phenotypic plasticity in response to food or space availability. In our experiment, juvenile *A. percula* had growth rates that were positively related to the size of their anemone hosts. This growth plasticity was not a response to variation in food availability alone, since we fed fish the same amounts of food. Further, growth plasticity was likely not a response to variation in space availability alone, since it did not occur when juveniles were paired with artificial anemones. It is likely that another biological aspect of the mutualism, such as egesta produced by the anemone or cues necessary for the fish to assess that they are in suitable habitat, caused the observed phenotypic plasticity.

One potential explanation for the results is that the egesta produced by the anemone provided nutrients not provided by the pellet food and that larger anemones provided more nutrients leading to increased growth rates. In the wild and in laboratory settings where anemones are fed heterotrophic food, anemones expel undigested waste products in the form of egesta. It is known that anemonefishes compete aggressively for and consume anemone egesta in the wild (personal observations) and recent studies have shown that anemone egesta provide a substantial and predictable source of nutrients such as carbon and nitrogen for anemonefishes^22^. However, in our experiments we did not feed anemones, though some fish food might have been consumed by anemones, and we did not observe any anemones producing egesta. Other members of our lab group have not observed this laboratory population of *A. percula* consuming egesta or this population of *E. quadricolor* producing egesta in hundreds of hours of video analysis (Barbasch and Pacaro, pers. comm. 2021). Therefore, it is unlikely that the anemonefish in experiment 1 received different nutrients from the fish in experiment 2. Some other biological characteristic of the real anemones that was not present in the artificial anemones must provide the cue for growth plasticity in anemonefish.

Another, more likely, explanation for our results is that the anemonefish only respond to available space in conjunction with cues that indicate suitable habitat, i.e., real anemones. We found that *A. percula* juveniles paired with artificial anemones had lower survival compared to those paired with real anemones, and those that did survive grew slower and had more variable growth rates than those paired with real anemones. This raises the question of whether the pairing with real anemones is necessary for the fish to develop normally. All 28 species of anemonefish are obligate mutualists with one or more species of sea anemone, never found without their hosts in the wild [see reviews in^15,20,45^]. Changes in the host anemone, such as bleaching due to thermal stress, can have cascading effects on anemonefishes, including enhanced stress and changes to reproduction, behavior, and metabolism^46,47^. Mortality rates in juvenile anemonefishes are often high in aquaria [^48^ and references therein]. Though the reasons are not always known, it seems quite likely that anemones provide cues necessary for typical development of juvenile anemonefishes, and that stress levels and metabolism rates are different in anemonefish lacking anemones, influencing survival and growth patterns as observed in our second experiment. (With the emergence of anemonefishes as a model system for eco-evo-devo (Roux et al. 2020), this is something investigators should be aware of, since many anemonefishes used for research purposes are raised without host anemones and this seems likely to influence results and conclusions of investigations).

Growth plasticity in response to the size of their mutualistic hosts is likely adaptive for anemonefishes, though more research is necessary. On the one hand, selection will favor fish that grow large, since larger size is correlated with lower predation rate and higher reproductive output^43,49,50^. On the other hand, selection will favor fish that do not become too large for the available resources, since being too large may expose them to predators^15^ and/or mean that they cannot sustain themselves on available resources^51^. *Amphiprion percula* are plankton feeders and restricted in their foraging area by the space provided by the protective anemone tentacles. It would therefore be adaptive for dominant breeders to adjust their growth rate based on the size of their mutualistic host. However, since the current study presents an aquarium experiment and we found a relatively small effect size for the impact of anemone area on fish growth, we must ask ourselves, how relevant are our findings when thinking about wild populations?

**Figure 3:**
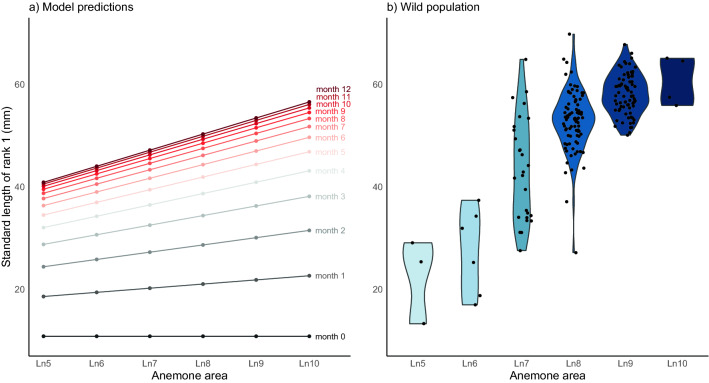
(**a**) Predicted sizes of rank 1 *Amphiprion percula* over 12 months in anemones of varying sizes (ln 5–ln 10 cm^2^). Using the mean probability estimates of the Bayesian mixed model analyses (Fig. 2a), change in SL per month was calculated as a function of initial SL and ln anemone area: ${\Delta SL}_{t0-1} ={initial\, SL}_{t0}+6.42+\left(0.81*\mathrm{ln}\left(anemone\,area\right)\right)-\left(0.25*{initial\, SL}_{t0}\right)$. (**b**) Distribution of rank 1 *A. percula* SL (mm) and the size of the anemone they reside in (binned, Ln cm^2^) from a population in Kimbe Bay, Papua New Guinea.

To answer this question, we used our model estimates to predict the sizes of anemonefish starting at the same size (10.86 mm, the average size of juveniles in experiment 1) and growing in anemones of different sizes for 12 months (until they asymptote). Using the mean probability estimates of the Bayesian mixed model analyses (Fig. 2a), we calculated change in SL per month as a function of initial SL and ln anemone area: ${\Delta SL}_{t0-1} ={initial\, SL}_{t0}+6.42+\left(0.81*\mathrm{ln}\left(anemone\, area\right)\right)-\left(0.25*{initial\, SL}_{t0}\right).$ The anemone sizes were chosen to be representative of the sizes of anemones we find with *A. percula* in the wild. The result is that we predict to find fish of significantly different sizes in different anemone sizes (Fig. 3a). Even with our simple model, only taking initial size and anemone size into account, the predicted fish sizes are within the range of those found in wild fish paired with anemones of the sizes used in the calculation (Fig. 3b). Small as the effect of anemone size on growth may seem, over the lifetime of an anemonefish, which can live for several decades^52^, adapting to be the ideal size for the available host may make a large difference.

While we provide the first experimental study of the phenomenon, phenotypic plasticity of growth in response to a mutualistic interaction is likely widespread in both vertebrates and invertebrates. Many mutualistic partners are size matched in the wild and often, like we observed in the clown anemonefish, the size of one partner appears to be a compromise between maximizing reproduction and dispersal, and not outgrowing their partner, stabilizing the mutualism. For example, fig pollinator wasps are more successful at dispersal when larger but the fig’s ostiole limits maximum wasp size^11^. In ant-plant mutualisms, plant growth rates and ant colony growth, as well as plant size and ant body size are often synchronized, suggesting positive feedback loops^53,54^. Phenotypic plasticity commonly mediates ecological interactions [reviewed by^55^] and may facilitate evolutionary matching between mutualistic partners^53,56–58^. More studies on mutualistic interactions involving vertebrates may shed light on how common growth plasticity and other forms of developmental plasticity are in this type of species interaction.

## Conclusion

In conclusion, we provide the first experimental evidence for vertebrate growth plasticity in response to a mutualistic interaction. We rule out food availability and space availability by itself as possible mechanisms. It is likely that space availability together with a biological cue from the mutualistic host are responsible for the pattern. The ability to anticipate the ideal body size in relation to the mutualistic partner is most likely present in other mutualistic relationships. More research is necessary to understand the intricate interplay of mutualistic partners and the effect on animal growth regulation and developmental phenotypic plasticity.

## Supplementary Information

Supplementary Information.

## Data Availability

Data will be made fully accessible on Dryad upon publication of the study.
