# Supplementary material for: Vertebrate growth plasticity in response to variation in a mutualistic interaction
Source: Sci Rep. 2022 Jul 18;12:11238. doi: 10.1038/s41598-022-14662-4 (PMC9293916; doi:10.1038/s41598-022-14662-4)
Supplement: Supplementary file 1 — Supplementary Information. [file 41598_2022_14662_MOESM1_ESM.docx]

Supplemental Material

Vertebrate growth plasticity in response to variation in a mutualistic interaction

**1. Survival and growth**

To test if survival probability differed between the two experiments (artificial vs real anemones) we conducted a Cox Proportional Hazard model using the *survival* package (Therneau & Grambsch, 2000; Therneau, 2021). We used survival (time and status) as dependent variable, anemone type (categorical: artificial, real) and initial fish standard length at the time of introduction as independent variables.

Survival was much lower for fish in artificial anemones compared to those paired with real anemones (Figure 1a). Being paired with an artificial anemone increased the hazard by a factor of 2.48 (95% CI: 1.41, 4.37, p=0.002). Larger juveniles also had a significantly higher probability of mortality, with the hazard increasing by a factor of 0.73 (95% CI: 0.65, 0.83, p<0.001). The interaction between anemone type and initial size was not significant (χ^2^=0.837, p=0.360).

Growth was much lower and more variable for fish in artificial anemones compared to those paired with real anemones (Figure 1b). Over the six months of the respective experiment, fish in real anemones had a mean (± standard error (s. e.)) growth of 22.51 ± 0.59 mm, growing to a mean (± s. e.) of 2.11 ± 0.09 times their initial size, while fish paired with artificial anemones only grew 15.89 ± 1.95 mm, 1.62 ± 0.22 times their initial size.


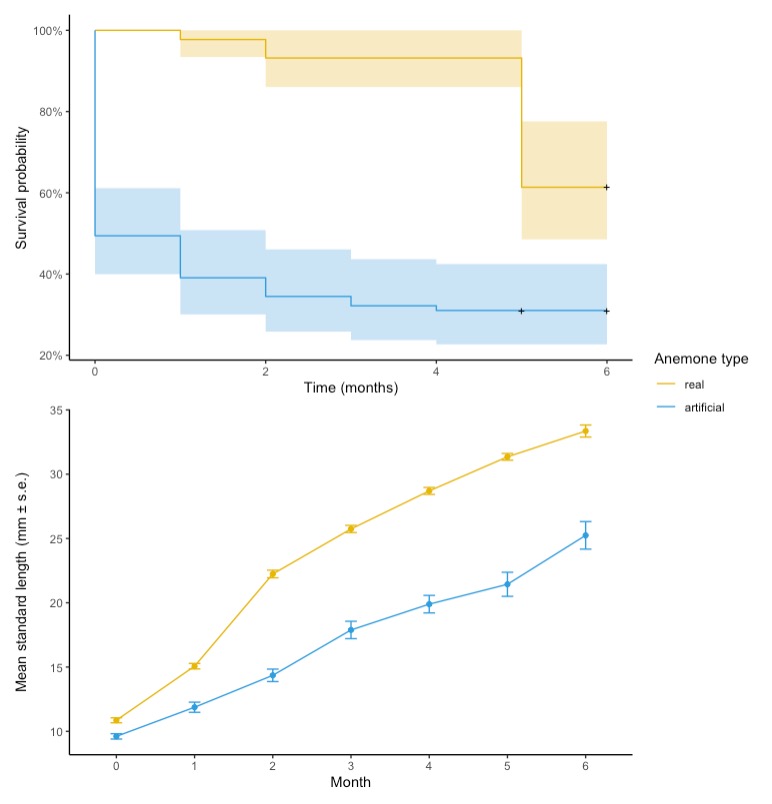


Figure 1. a) Survival probability (± 95% confidence intervals) for *Amphiprion percula* in experiment 1 (n_initial_=44, paired with *Entacmaea quadricolor*) and experiment 2 (n_initial_=88, paired with artificial anemones) over 6 months; b) Mean standard length (mm ± standard error (s. e.)) of *Amphiprion percula* in experiment 1 and experiment 2 over 6 months.

**2. Model diagnostics**


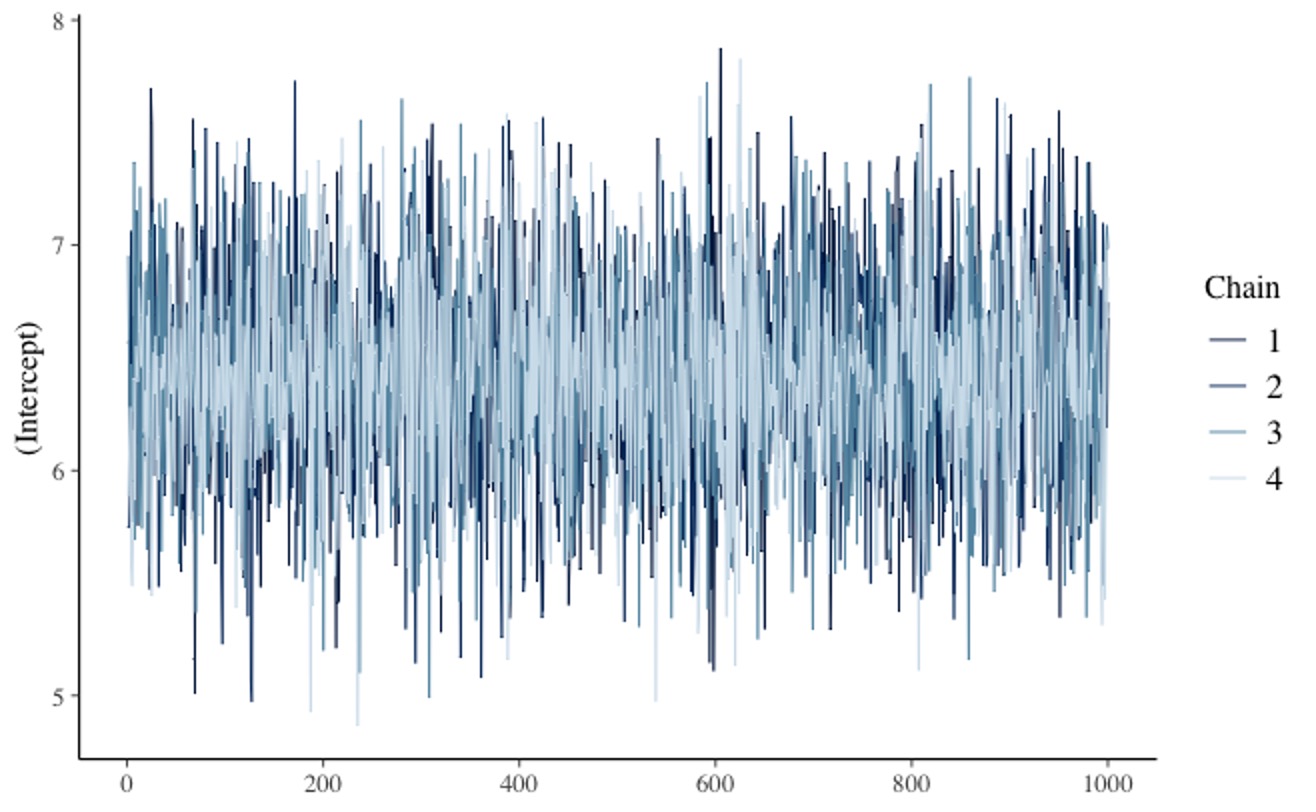


Figure 2. Trace plot for the Bayesian mixed model from experiment 1, testing the effect of Ln anemone area and initial standard length on the growth of *Amphiprion percula*.


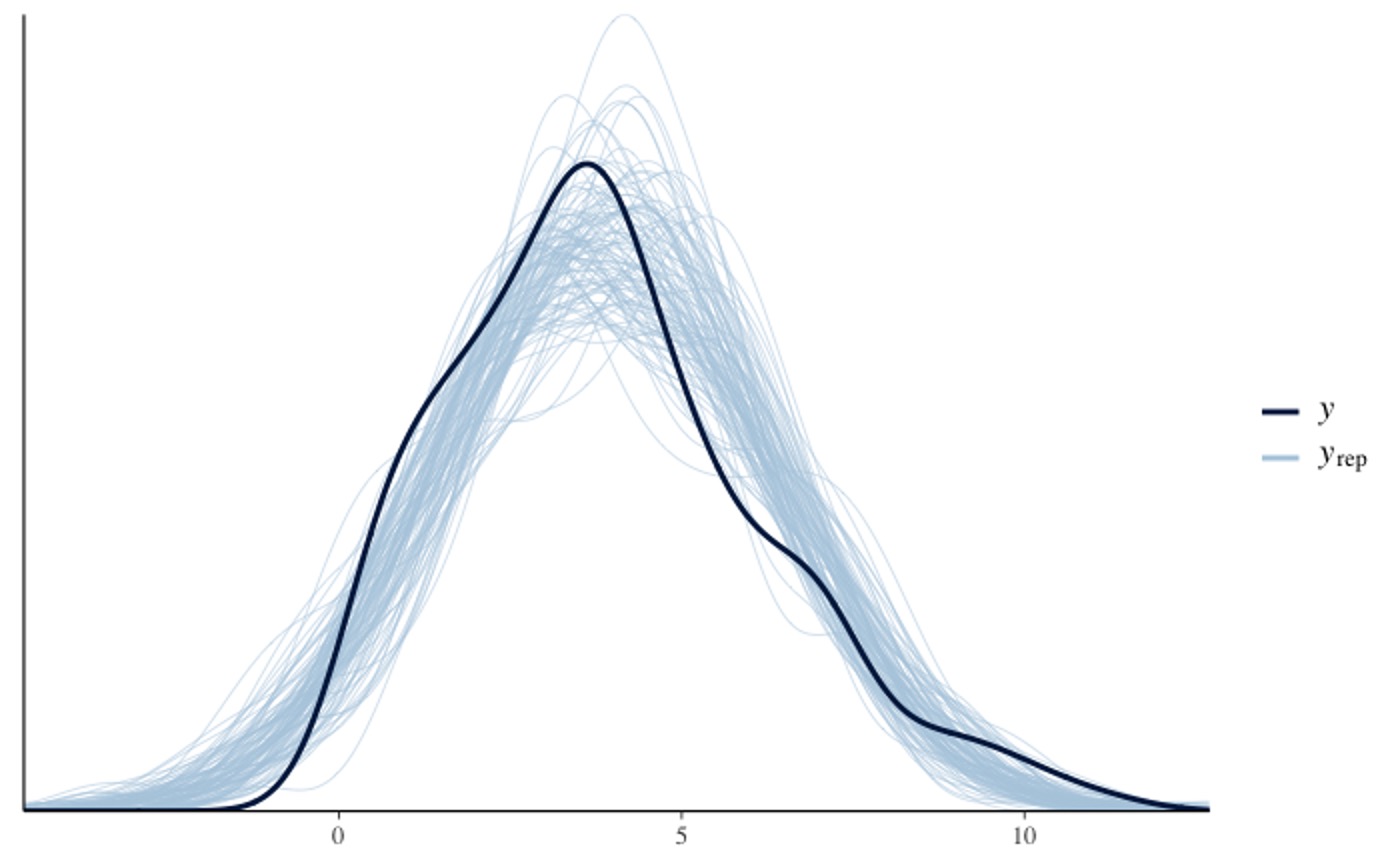


Figure 3. Kernel density estimate for the Bayesian mixed model from experiment 1. Plotted are the observed data set *y* (dark curve), with density estimates for 100 simulated data sets y_rep_ drawn from the posterior predictive distribution (light curves).


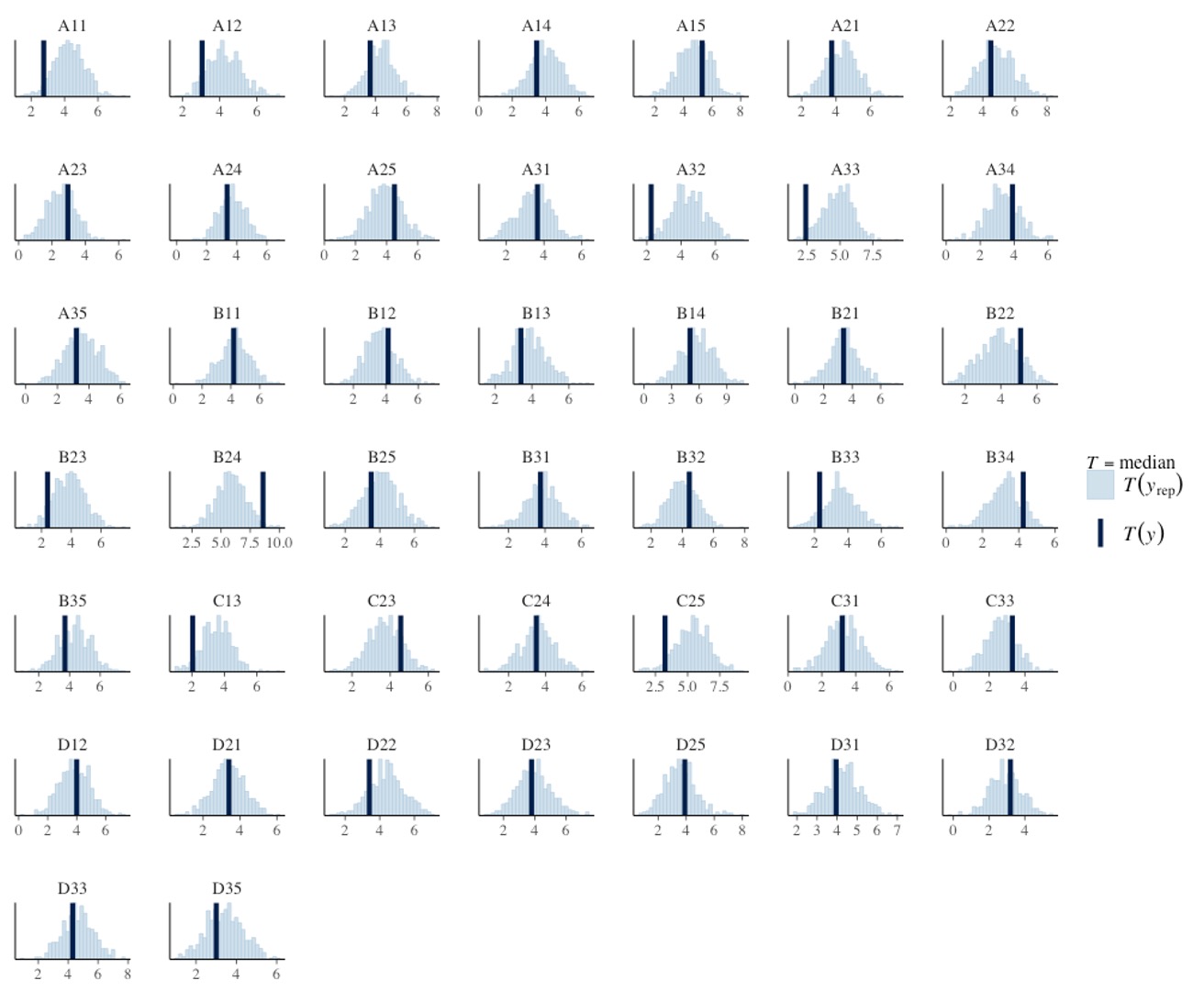


Figure 4. Histogram of statistics for experiment 1 computed from 500 draws from the posterior predictive distribution for each individual fish. The dark vertical line is computed from the observed data.


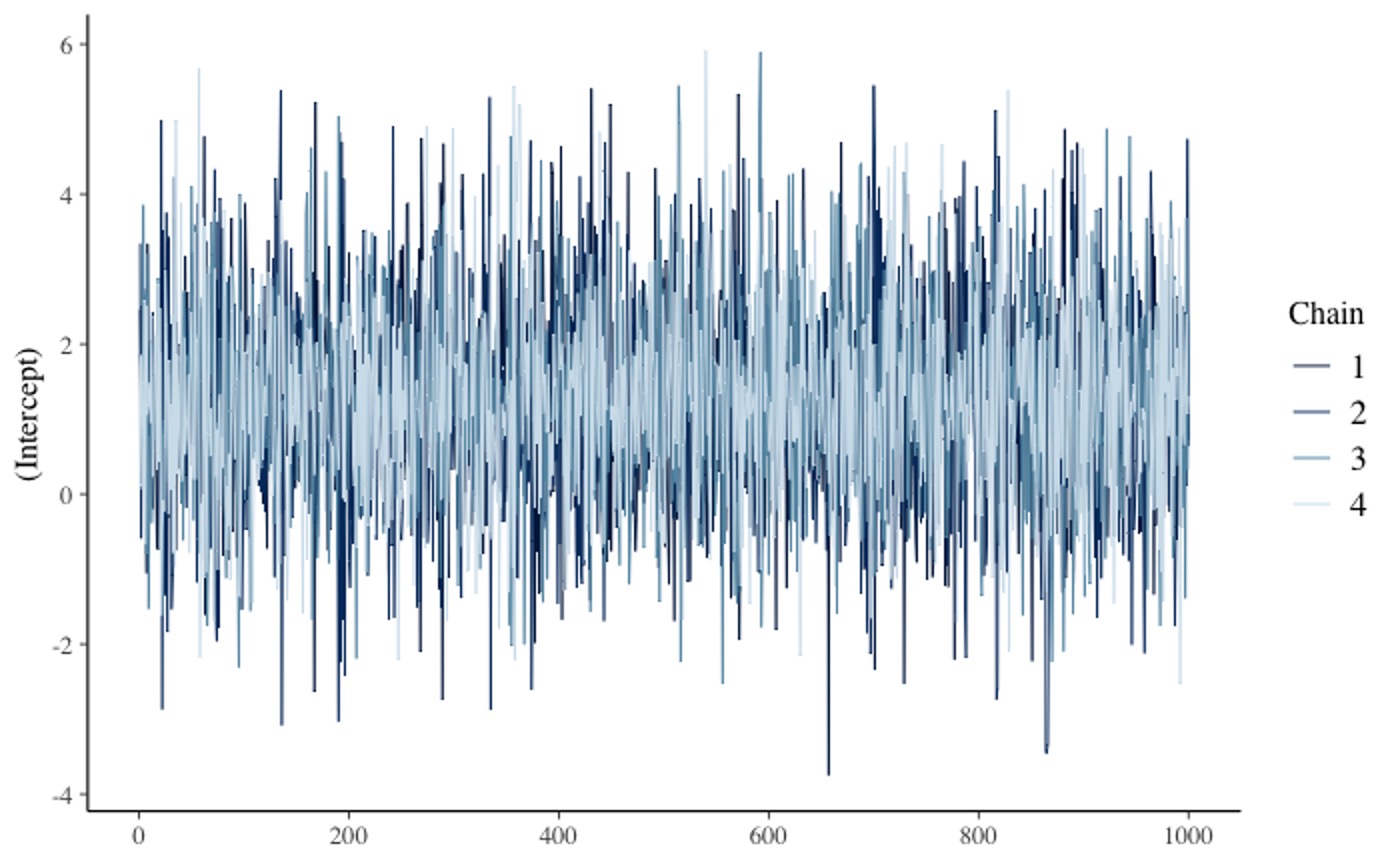


Figure 5. Trace plot for the Bayesian mixed model from experiment 1, testing the effect of Ln anemone area and initial standard length on the growth of *Amphiprion percula*.


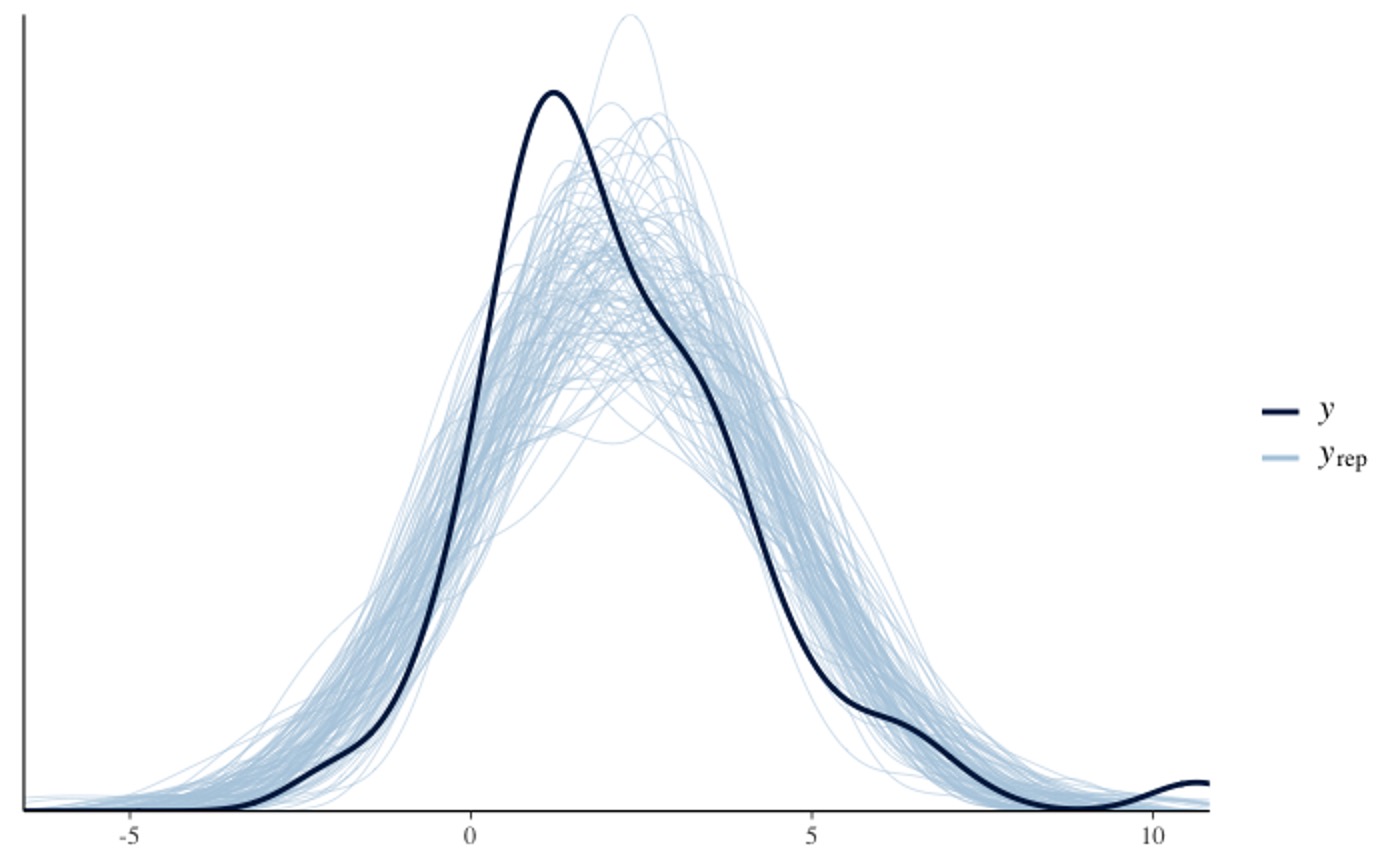


Figure 6. Kernel density estimate for experiment 2 of the observed data set *y* (dark curve), with density estimates for 100 simulateddata sets y_rep_ drawn from the posterior predictive distribution (light curves).


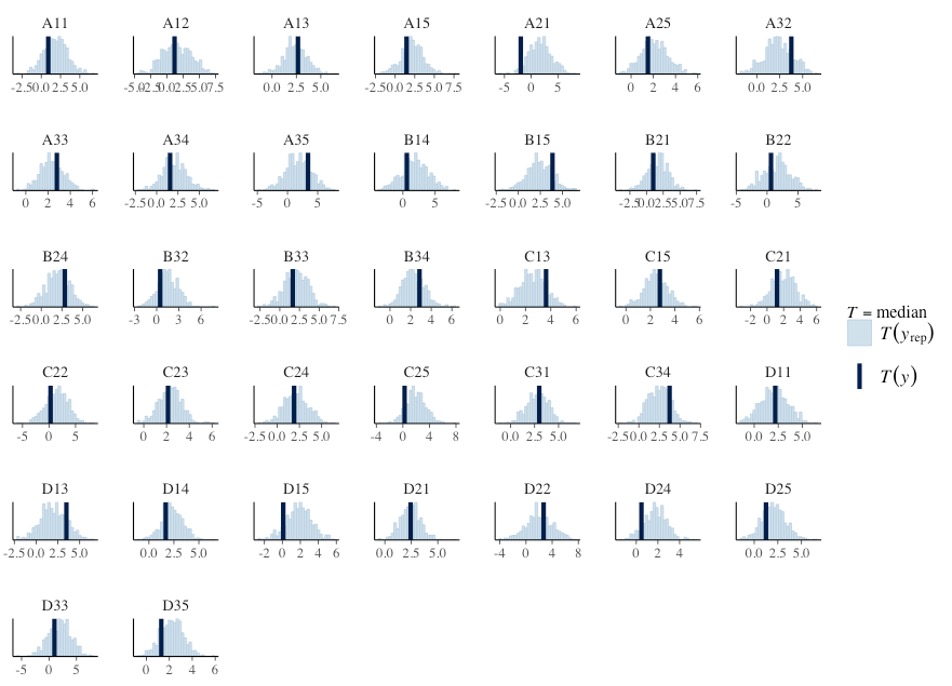


Figure 7. Histogram of statistics for experiment 2 computed from 500 draws from the posterior predictive distribution for each individual fish. The dark vertical line is computed from the observed data.
